# Photorespiratory Engineering from an Evolutionary Perspective: A Constraint-Aligned Evaluation and Validation Framework

**DOI:** 10.3390/biology15211964

**Published:** 2026-11-01

**Authors:** Wan-Ting Huang, Wen-Hui Liao, Xiao-Yi Qin, Jun-Jie Zhu

**Affiliations:** Guangxi Key Laboratory of Forest Ecology and Conservation, College of Forestry, Guangxi University, Nanning 530004, China

**Keywords:** C_3_ crops, Rubisco, bypass engineering, evolutionary constraints, constraint-aligned framework, validation pipeline

## Abstract

More than two billion years of photorespiratory evolution have shaped three sets of constraints: catalytic constraints associated with Rubisco oxygenase activity; compartmental topology constraints arising from compartmental organization, transport processes, and cofactor coupling; and physiological and ecological constraints arising from integration with metabolic and signaling networks. Photorespiratory engineering has emerged as a key strategy for improving photosynthetic efficiency in C_3_ crops, yet existing studies have not systematically assessed the practical implications of these constraints. Evaluation metrics remain inconsistent, the scope and depth of stability and risk assessments vary, and methodological heterogeneity hinders comparisons across studies. To address these issues, we synthesize the evolutionary history of photorespiration and findings from previous research to develop an evaluation and validation framework aligned with these constraints. This framework comprises eight assessment dimensions defining what to report, a context matrix specifying the conditions under which assessments should be conducted, and a five-tier validation pipeline defining the sequence of validation steps and the criteria for classifying outcomes. Retrospective application to five published case studies demonstrates that the framework enables consistent classification of outcomes and identifies distinct priorities for further improvement across studies involving different crop species, engineering strategies, and evaluation endpoints. By providing a practical evidence structure that can be updated as new findings emerge, this framework supports the transition from empirical exploration to systematic photorespiratory engineering and facilitates cross-study comparisons and the cumulative synthesis of evidence across assessment dimensions, experimental contexts, and outcomes.

## Simple Summary

Photorespiration consumes energy and can limit growth and yield in many crops. Photorespiration has evolved over more than two billion years, leaving three enduring constraints on its redesign: the inherent catalytic limitations of Rubisco, the complexity of a pathway spanning three cellular compartments, and its extensive integration with whole-plant metabolism. Although photorespiratory engineering aims to increase crop yields, outcomes vary widely, and a standardized framework for evaluating its effectiveness is lacking. By examining these evolutionary constraints, this review develops a common evaluation framework comprising eight key assessment areas, ranging from the expression of engineered genes and metabolic flux to final yield and the stability of performance under field conditions, and five progressively more demanding levels of evidence, from molecular characterization to rigorous multilocation field trials, to facilitate comparisons across studies. Application of this framework to five published case studies identifies both well-supported findings and gaps in the available evidence. A shared checklist facilitates the verification of findings and helps researchers build on previous work, thereby accelerating the development of more productive crops to meet the needs of a growing global population.

## 1. Introduction

In oxygenic photosynthetic organisms, ribulose-1,5-bisphosphate carboxylase/oxygenase (Rubisco) catalyzes both the carboxylation and oxygenation of ribulose-1,5-bisphosphate (RuBP), with the latter reaction producing equimolar amounts of 3-phosphoglycerate (3-PGA) and 2-phosphoglycolate (2-PG) [1,2]. Because 2-PG cannot directly enter the Calvin–Benson cycle and inhibits several key metabolic reactions in chloroplasts [2,3], it must be metabolized via the photorespiratory pathway, also known as the C_2_ cycle, which involves multiple enzymes distributed across chloroplasts, peroxisomes, and mitochondria [1,2]. The canonical C_2_ cycle recovers three of the four carbon atoms in two molecules of 2-PG as one molecule of 3-PGA, releasing the remaining carbon atom as CO_2_. Together with the reassimilation of the NH_3_ and CO_2_ released during the cycle, this process imposes substantial demands for ATP and reducing equivalents, thereby limiting net carbon assimilation and the yield potential of C_3_ crops, particularly under conditions of high temperature or low intercellular CO_2_ concentration [4]. Beyond its role in carbon salvage, photorespiration is closely integrated with plant physiology. It serves as an alternative electron sink that helps maintain redox balance under fluctuating light, contributes to nitrogen recycling through ammonia reassimilation, interacts with sulfur assimilation, and is closely linked to one-carbon metabolism and source–sink signaling networks. Photorespiration is therefore considered a key target for engineering improvements in photosynthetic efficiency in C_3_ crops [5,6,7].

Over the past two decades, researchers have sought to reduce the carbon and energy costs of photorespiration by engineering Rubisco, introducing carbon-concentrating mechanisms (CCMs), and redesigning 2-PG salvage pathways [8,9,10]. Improvements in photosynthesis, biomass accumulation, or yield have been reported in *Arabidopsis* [11], tobacco [9], rice [12,13], and potato [14], highlighting the potential of photorespiratory engineering to improve crop performance. These advances encompass diverse engineering strategies, including a complete decarboxylation bypass (GMA in rice) [15], partial decarboxylation bypasses (the Kebeish pathway in *Arabidopsis* [11] and the glycolate:glyoxylate aminotransferase (GCGT) bypass in rice [13]), a cytosolic bypass (BHAC in *Arabidopsis*) [16], and complete replacement of native Rubisco in tobacco [8]. However, these studies differ substantially in genetic background, engineering strategy, environmental conditions, and evaluation endpoints. Furthermore, source–sink coordination constrains the relationships among photosynthetic rate, biomass accumulation, and yield [17,18], precluding simple linear extrapolation from one endpoint to another. This heterogeneity complicates comparisons of the magnitude and stability of reported gains and the identification of their underlying mechanisms. Consequently, evaluating these interventions requires addressing several questions beyond whether they are effective: Which mechanisms underlie the observed gains? Under what conditions do these mechanisms operate? Can improvements at the leaf level translate into reproducible yield gains, and under what conditions? Do these interventions impose hidden metabolic or reproductive costs? These questions reflect the challenges of engineering a complex system shaped by long-term evolution. Examining its evolutionary history can help explain the origins of these challenges, why individual experiments or metrics cannot adequately address them, and where engineering interventions may encounter constraints, incur risks, or reveal opportunities. We use the term “flexible constraints” to describe evolutionarily shaped features that constrain engineering outcomes but can be reconfigured under appropriate conditions, distinguishing them from fixed limits imposed by thermodynamics or stoichiometry.

The evolutionary history of photorespiration is closely linked to that of photosynthesis, as Rubisco oxygenase activity is an intrinsic consequence of its catalytic chemistry [2,19]. Photorespiratory flux was likely low under the low-O_2_, high-CO_2_ conditions of the early atmosphere. The Great Oxidation Event (GOE), approximately 2.4–2.3 billion years ago, marked a major rise in atmospheric O_2_ and preceded the origin of plastids [20,21]. Primary endosymbiosis subsequently introduced the cyanobacterial ancestor of plastids, together with Rubisco, into a eukaryotic host. Although cyanobacterial CCMs and the evolutionary loss of carboxysome-related genes have been implicated in the emergence of photorespiration in the green lineage, their timing relative to plastid acquisition remains uncertain [22]. Over evolutionary time, rising O_2_ availability and declining CO_2_ availability increased the selective pressure to metabolize the products of Rubisco oxygenation. The recruitment and reorganization of metabolic pathways in the host and endosymbiont contributed to the establishment of a photorespiratory pathway spanning chloroplasts, peroxisomes, and mitochondria, giving rise to the three-compartment organization characteristic of land plants [2,23].

Three sets of evolutionarily shaped flexible constraints correspond to three major challenges in photorespiratory engineering: catalytic constraints help explain the difficulty of simultaneously improving Rubisco specificity and catalytic efficiency; compartmental constraints help explain why bypass designs can lead to byproduct accumulation and imbalances in energy and reducing equivalents; and physiological and ecological constraints help explain why improvements at the leaf level do not consistently translate into yield gains. At the catalytic level, oxygenase activity is intrinsic to Rubisco’s catalytic mechanism, and changes in CO_2_/O_2_ specificity can involve trade-offs among carboxylation efficiency, substrate affinity, and protein stability [24,25]. At the compartmental level, reactions in the three-compartment pathway are coordinated with metabolite transport, cofactor supply, and byproduct removal; modifying individual reactions in isolation can therefore disrupt pathway flux and metabolic balance [26,27]. At the whole-plant and ecological levels, photorespiration is integrated into networks involving redox buffering, carbon, nitrogen, and sulfur metabolism, source–sink development, and stress responses [17,28,29,30], making engineering outcomes highly dependent on genetic background and environmental conditions. Interventions that depart more substantially from the native network may therefore carry a greater risk of adverse effects across multiple aspects of plant performance.

Recent reviews of photorespiratory engineering have broadly followed three lines of inquiry: classifying engineering strategies by bypass type and carbon fate [31], evaluating bypass energetics and flux characteristics [32], and examining the role of photorespiration as a metabolic hub [33]. These reviews have addressed what to engineer, whether the energy balance is favorable, and how photorespiration is integrated into the broader metabolic network. However, they have not fully integrated the mechanistic attribution of gains, the conditions under which those gains occur, their translation from the leaf level to crop yield, and potential hidden metabolic or reproductive costs into a common evaluation framework. Thus, although existing reviews provide a systematic foundation for selecting engineering targets, a unified framework is still needed to connect evolutionarily shaped constraints across biological scales with engineering design choices, evidence-reporting requirements, and decisions at successive stages of validation.

This review adopts an evolutionary perspective on photorespiration, first examining how catalytic, compartmental, and physiological and ecological features impose flexible constraints and under what conditions these constraints can be reconfigured. This evolutionary analysis identifies functional requirements that engineering designs must accommodate and helps define the conditions under which these designs can operate successfully. These requirements inform an evaluation and validation framework aligned with evolutionary constraints, comprising eight assessment dimensions, a G×E×D×M×S context matrix encompassing genetic background (G), environment (E), engineering design (D), management practices (M), and source–sink status and developmental stage (S), and a five-tier validation pipeline. These components specify, respectively, what to measure, the contexts in which measurements should be made, and the sequence of validation steps needed to build evidence for the proposed causal relationships. We then retrospectively apply the framework to five case studies selected to assess its applicability across diverse engineering strategies and levels of evidence: the GMA bypass, the Kebeish pathway, the GCGT bypass, the BHAC bypass, and complete Rubisco replacement. Specifically, we assess the extent to which each study addresses the eight assessment dimensions, characterizes the relevant G×E×D×M×S contexts, and provides evidence corresponding to the H1–H5 validation tiers. Drawing on this analysis, we discuss the framework’s scope, limitations, and priorities for further development, with the aim of supporting consistent evaluation, evidence accumulation, and iterative refinement in photorespiratory engineering research.

## 2. Formation, Characteristics, and Engineering Implications of the Three Classes of Photorespiratory Constraints

**Figure 1:**
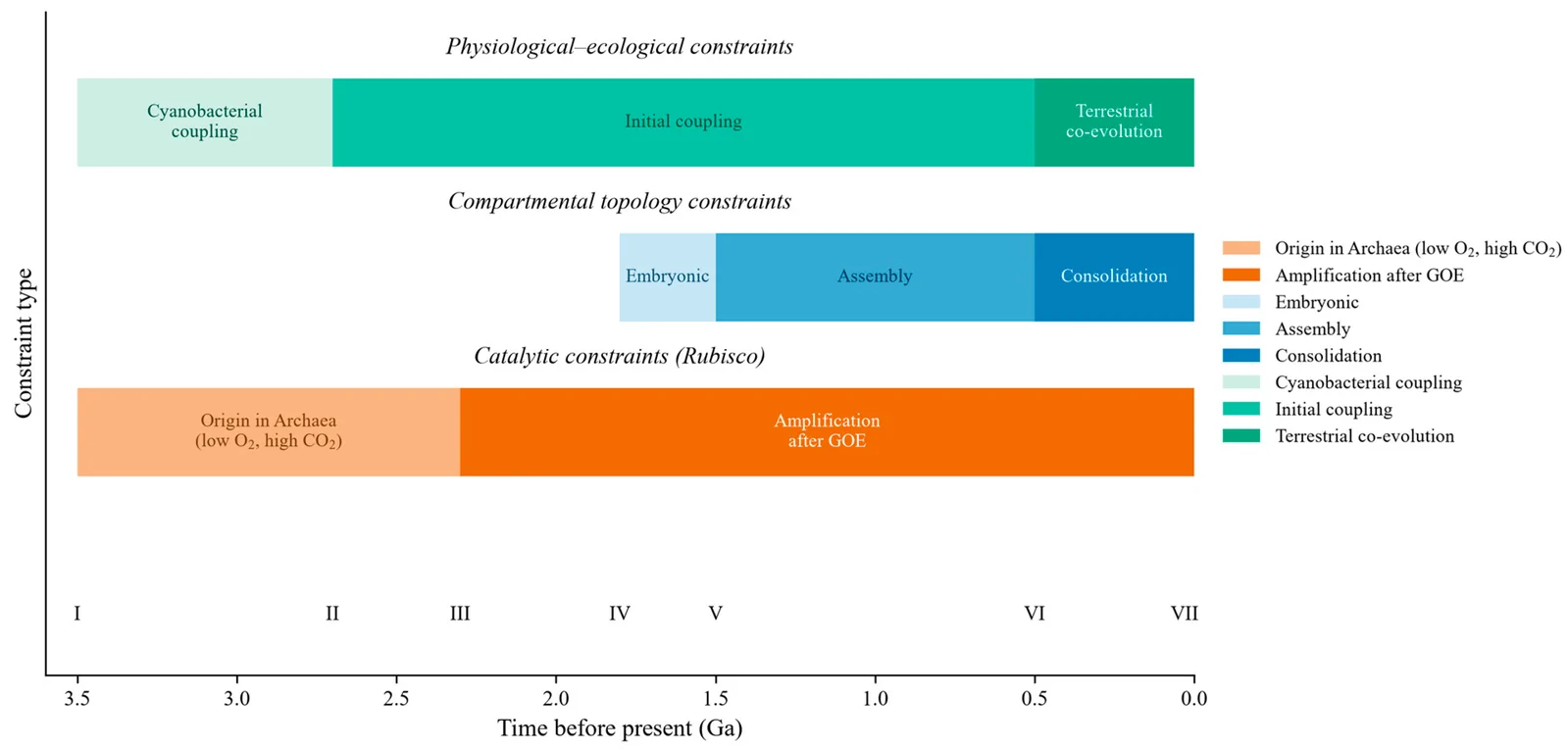
Evolutionary timeline of photorespiratory constraints. The band representing physiological and ecological constraints extends back to the inferred origin of oxygenic photosynthesis, reflecting the early coupling of photorespiratory metabolism to environmental conditions and cellular metabolic networks in cyanobacteria, which also possess photorespiratory pathways. Very light shading indicates uncertainty in the timing of this early phase (~3.5–2.7 billion years ago, Ga). Tick marks indicate approximate dates of major evolutionary events: I, proposed archaeal origin of an ancestral Rubisco lineage (~3.5 Ga); II, diversification of crown-group cyanobacteria (~2.7 Ga); III, the Great Oxidation Event (~2.3 Ga); IV, mitochondrial endosymbiosis and the subsequent emergence of the last eukaryotic common ancestor (~1.8 Ga); V, primary plastid endosymbiosis (~1.5 Ga); VI, colonization of land by plants, in which the three-compartment photorespiratory pathway is characteristic (~0.5 Ga); and VII, repeated independent origins of C_2_ and C_4_ photosynthesis, represented schematically at ~30 million years ago (Ma). Dates are approximate, and several events, particularly the origins of Rubisco and oxygenic photosynthesis and the timing of endosymbioses, remain uncertain.

The evolutionary emergence of the three classes of constraints is outlined in Figure 1.

These constraints share four features: (1) evolutionary origins, reflecting the long-term evolution of photorespiration through natural selection; (2) quantifiable boundaries that can be characterized using measurable relationships, capacities, or fluxes, such as the covariation between Rubisco CO_2_/O_2_ specificity (S_C/O) and carboxylation turnover rate (k_cat,C), H_2_O_2_ scavenging capacity, and NH_3_ reassimilation flux; (3) conditional flexibility, allowing some boundaries to shift through appropriate engineering designs or favorable environmental conditions; and (4) potentially increasing costs as interventions depart further from the native system, with a greater risk of adverse effects across multiple aspects of plant performance. In the following sections, we trace the evolutionary origins and key characteristics of each class of constraints, examine their implications for engineering, and discuss how these implications are reflected in existing efforts to engineer crop photorespiration.

### 2.1. Catalytic Constraints of Rubisco

#### 2.1.1. Evolutionary Origin

The Rubisco superfamily comprises four major forms (I–IV), whose evolutionary relationships have been interpreted as supporting an archaeal origin of an ancestral Rubisco gene [34]. Forms I–III catalyze both carboxylation and oxygenation, whereas Form IV comprises Rubisco-like proteins (RLPs) that generally catalyze neither reaction [35]. Horizontal gene transfer and subsequent diversification contributed to the distribution of Rubisco across prokaryotic lineages, with Form I becoming the predominant form in oxygenic photosynthetic organisms. Estimates place the diversification of crown-group cyanobacteria at approximately 2.7–2.2 Ga [36] and the emergence of the last eukaryotic common ancestor (LECA) at approximately 1.8 Ga [37,38]. These estimates are consistent with the presence of Rubisco in cyanobacteria before the emergence of LECA, although they do not establish its timing relative to the earliest eukaryotic hosts. Rubisco oxygenation is an intrinsic side reaction whose physiological cost depends partly on the CO_2_/O_2_ ratio at the active site. Evolutionary changes in Rubisco form and provenance also illustrate the potential for enzyme replacement. For example, some dinoflagellates possess plastid-localized, nucleus-encoded Form II Rubisco of bacterial ancestry [39,40], while phylogenetic analyses have implicated horizontal gene transfer from α-proteobacteria in the evolutionary history of Form ID Rubisco in certain non-green algal lineages [41]. Such changes can require coordinated modifications in gene expression, protein targeting, and metabolic regulation.

#### 2.1.2. Formation and Nature of Catalytic Flexible Constraints

Rubisco’s discrimination between CO_2_ and O_2_ does not primarily depend on substrate recognition through a conventional binding step. Instead, it arises largely from differences in the activation free energies of their reactions with the ribulose-1,5-bisphosphate (RuBP) enediolate intermediate, including differences in transition-state stabilization [19,42]. Consequently, modifying active-site discrimination between the two gases can also affect other catalytic steps, although the structural basis of these effects remains incompletely understood [43]. Several kinetic parameters of naturally occurring Rubiscos covary, including CO_2_/O_2_ specificity (S_C/O), carboxylation turnover rate (k_cat,C), the Michaelis constants for CO_2_ and O_2_ (K_C and K_O), and oxygenation turnover rate (k_cat,O) [44]. These relationships are more complex than a simple inverse correlation between S_C/O and k_cat,C. For example, Rubiscos from C_4_ plants generally exhibit higher carboxylation turnover rates and lower S_C/O values than those from C_3_ plants, consistent with the coevolution of Rubisco kinetics and CO_2_-concentrating mechanisms (CCMs) [24,25]. Rubisco performance also depends on reaction chemistry [19], evolutionary history [45], protein folding and assembly [46], activation state [47], and the local CO_2_ environment [24]. Together, the intrinsic oxygenase reaction and the interdependence of catalytic parameters define a set of constraints whose practical consequences depend on the enzyme’s cellular and environmental context. Departures from a native combination of kinetic parameters may therefore introduce trade-offs, but their effects must be evaluated in the context of the host system. Complete replacement with a heterologous Rubisco presents a particularly demanding test of compatibility: depending on the Rubisco form and replacement strategy, successful implementation may require compatible folding and assembly machinery, including factors such as RAF1, RAF2, and BSD2, coordinated expression and stoichiometric assembly of the constituent subunits, and compatibility with Rubisco activase [46,47]. If the introduced enzyme has lower CO_2_/O_2_ specificity than the native enzyme, the ratio of oxygenation to carboxylation may increase at a given CO_2_/O_2_ ratio. The net physiological outcome therefore depends on the interaction between enzyme kinetics and the chloroplast CO_2_ environment, rather than on carboxylation turnover alone. The occurrence of low-specificity Form II Rubisco in dinoflagellates illustrates that such enzymes can support photosynthesis in an appropriate cellular context, potentially aided by CCMs or other mechanisms that modify the local CO_2_/O_2_ ratio; however, this observation alone does not establish which compensatory mechanisms are required [39].

#### 2.1.3. Engineering Implications

The Rubisco catalytic constraints discussed in this section make Rubisco itself an upstream target for photorespiratory engineering: reducing its oxygenation rate could decrease 2-PG production at the source. For variants claimed to improve S_C/O, k_cat,C, and carboxylation efficiency simultaneously, a full set of kinetic parameters should be measured independently, together with the enzyme’s activation state, assembly efficiency, and abundance in leaves; these measurements should be made under physiologically relevant temperature and CO_2_/O_2_ conditions. Complete-replacement designs also require assessment of compatibility between the heterologous enzyme and the host’s assembly chaperones and Rubisco activase, as well as whether the chloroplast CO_2_ environment can compensate for differences in substrate specificity. The evolutionary relationship between Rubisco and Rubisco-like proteins (RLPs) further suggests that catalytic functions are not immutable. The evolution of Rubisco-mediated carbon fixation from an ancestral protein related to RLPs—which include proteins involved in sulfur metabolism and other core metabolic processes [34,48]—illustrates the potential for gain-of-function evolution. Although this makes the exploration of novel catalytic combinations conceivable in principle, translating that possibility into an engineering strategy remains challenging because of the substantial screening and protein-assembly requirements.

#### 2.1.4. Empirical Validation

Direct engineering of plant Rubisco has long been constrained by difficulties in protein folding and assembly and by limitations of available screening systems. Recent advances in chaperone engineering, including directed evolution of the Rubisco assembly chaperone RAF1, have improved the assembly of heterologous Rubisco. However, these experiments have primarily been conducted in Escherichia coli and have not yet demonstrated improved catalytic performance in planta [49]. By comparison, some engineered cyanobacterial Rubisco variants have shown increased carboxylation efficiency without compromising CO_2_/O_2_ specificity [24,25]. Complete replacement of native Rubisco has also been explored. In one study, *Halothiobacillus neapolitanus* Rubisco (HnRubisco) was introduced into tobacco chloroplasts to replace native Rubisco [8]. The introduced subunits assembled into functional L_8_S_8_ complexes that accumulated to approximately 40% of wild-type Rubisco levels. HnRubisco exhibited approximately twice the carboxylation turnover rate of tobacco Rubisco but approximately eightfold lower apparent CO_2_ affinity, and the transgenic plants required CO_2_ enrichment to 1% to sustain autotrophic growth. An alternative strategy modifies the catalytic properties of an existing Rubisco scaffold through amino acid substitutions. In another study, the C329A and A332V substitutions, informed by red algal loop 6 sequences, were introduced into *Rhodospirillum rubrum Rubisco* (RsRubisco), increasing k_cat,C by 60% and S_C/O by 7% [10]. Transgenic tobacco expressing the modified enzyme showed up to twofold increases in photosynthesis and growth relative to control plants expressing unmodified RsRubisco. Modifying an established scaffold may reduce assembly compatibility challenges relative to introducing a more distantly related enzyme, although the attainable improvements remain constrained by interactions among catalytic properties and by the structural context of the substitutions.

#### 2.1.5. Assessment of Evidence Completeness

Taken together, these examples show that cyanobacterial Rubisco mutants provide evidence that the catalytic step itself can be improved, although they represent only prokaryotic systems. Engineering of Raf1 indicates that some bottlenecks in Rubisco assembly can be overcome; however, it has not yet directly targeted Rubisco catalysis. To date, empirical improvements in the catalytic performance of plant chloroplast Rubisco have not been demonstrated. Studies involving complete Rubisco replacement have so far been conducted only under controlled conditions and have not yet been validated in the field; evidence regarding the compatibility of heterologous enzymes with plant Rubisco assembly and activation systems remains limited. HnRubisco can assemble in the absence of homologous chaperones, but it accumulates to only 40% of wild-type levels. Whether chloroplast CO_2_ concentrations can compensate for differences in Rubisco specificity has not been tested.

### 2.2. Compartmental Topology Constraints

#### 2.2.1. Evolutionary Origin

The three-compartment organization of photorespiration arose through two endosymbiotic events and the evolutionary integration of peroxisomes. The evolutionary origin of peroxisomes remains unresolved, although they are generally regarded as host-derived organelles [50,51]. Some hypotheses link their emergence to responses to oxidative stress following mitochondrial endosymbiosis [52]. Mitochondrial endosymbiosis occurred in the early Proterozoic (~1.8 Ga), establishing the energetic foundation for the last eukaryotic common ancestor (LECA) [37,53]. By that time, atmospheric O_2_ levels had already risen following the Great Oxidation Event (GOE), although the oceans remained largely anoxic [21]. Plastid endosymbiosis occurred later, approximately 1.5 Ga, and involved a cyanobacterial ancestor from a freshwater lineage [54,55]. Following endosymbiosis, most genes of the plastid ancestor were either lost or transferred to the host nuclear genome [56,57]. To enable the targeting of nucleus-encoded proteins to plastids, the host co-opted bacterial components, including homologs of Tic22 and Tic20, and evolved the TOC/TIC protein import machinery spanning the two envelope membranes [58,59]. These changes were accompanied by the coordination of gene expression between the nuclear and plastid genomes. In parallel, the host expanded its repertoire of endogenous transporters to facilitate the exchange of metabolites and energy carriers [60,61]. Nucleus-encoded plastid precursor proteins are recognized by their N-terminal transit peptides and imported into the stroma via the TOC/TIC machinery; some are subsequently directed to other plastid subcompartments, including the thylakoids [62,63]. Consequently, three organelles with distinct evolutionary origins became integrated into a single pathway: chloroplasts export glycolate, peroxisomes oxidize it, and mitochondria decarboxylate glycine. The ammonium released during glycine decarboxylation is returned to chloroplasts for reassimilation. This compartmental organization has been retained in land plants since their colonization of terrestrial environments [23,64,65]. The photorespiratory enzyme system itself has mosaic evolutionary origins: some enzymes derive from cyanobacteria, the ancestors of chloroplasts; others derive from heterotrophic bacteria, the ancestors of mitochondria; and phosphoglycolate phosphatase derives from archaea [22,26]. Thus, the modern three-compartment pathway did not arise from a single lineage but was progressively assembled from enzyme systems of different evolutionary origins, likely during early algal diversification [22].

#### 2.2.2. Formation and Nature of Compartmental Topology Flexible Constraints

As described above, the distribution of photorespiration across three organelles in extant land plants reflects progressive assembly over evolutionary time rather than deliberate design. From bryophytes to angiosperms, the canonical photorespiratory pathway spans chloroplasts, peroxisomes, and mitochondria, with core enzymes—including glycine decarboxylase (GDC), serine hydroxymethyltransferase (SHMT), glycolate oxidase (GOX), and catalase (CAT)—localized to their respective compartments [66]. Comparative studies spanning liverworts to angiosperms support a conserved role for photorespiration as an electron sink in land plants [67]. The integration of photorespiration into these organelles relied on pre-existing transport, cofactor, and reactive oxygen species (ROS)-scavenging systems. Consequently, photorespiratory flux across compartments is constrained by transporter capacity, the balance between cofactor supply and demand, and ROS-scavenging capacity. Beyond these three core organelles, the cytosol provides an important route for metabolite exchange; the vacuole may contribute to glycerate storage [68]; the redox state of the endoplasmic reticulum may indirectly influence mitochondrial GDC activity [69]; and a cytosolic glyoxylate shunt has been implicated in photorespiration-related metabolism [70]. The precise contributions of these additional compartments and processes to canonical photorespiration remain incompletely resolved and are therefore outside the scope of the core compartmental topology constraints discussed here.

The effective operation of the photorespiratory network requires coordination among metabolite transport, cofactor metabolism, and ROS scavenging. Together, these processes impose context-dependent constraints on compartmental reorganization, operating primarily at three levels. (1) Limited transport capacity. Photorespiratory carbon recycling involves multiple transport steps, but only a small number of the transporters involved have been experimentally characterized. Loss of the chloroplast glycolate/glycerate transporter PLGG1 causes glycolate and glycerate accumulation, reduces net photosynthesis, and produces a phenotype that is alleviated by elevated CO_2_ [71]. (2) Compartment-specific imbalances between cofactor and energy supply and demand. During glycine decarboxylation, the mitochondrial GDC generates NADH and transfers a one-carbon unit to tetrahydrofolate (THF), forming 5,10-methylene-THF, whereas the chloroplast Calvin–Benson cycle consumes ATP and NADPH. Coordination of reducing-equivalent transfer and carbon-skeleton exchange between compartments depends on specific shuttle and transport systems. Disruption of this coordination can prevent the network from maintaining balanced fluxes even when individual enzyme activities remain intact [2,72]. (3) Compartment-specific byproduct disposal. In peroxisomes, GOX-mediated glycolate oxidation produces one molecule of H_2_O_2_ per molecule of glycolate oxidized, placing H_2_O_2_ production in a compartment with high catalase activity. Chloroplast H_2_O_2_ scavenging instead relies on systems that include ascorbate peroxidases, which differ from catalase in H_2_O_2_ affinity, catalytic properties, and dependence on reductant supply [73,74,75]. Relocating GOX-mediated glycolate oxidation to chloroplasts may therefore generate H_2_O_2_ at rates that exceed the capacity of local antioxidant systems.

#### 2.2.3. Engineering Implications

Constraints imposed by compartmental organization do not imply that existing compartments are immutable; rather, any reorganization must satisfy four requirements: adequate substrate and product transport, balanced cofactor supply and demand, balanced carbon and nitrogen budgets, and sufficient removal of reactive by-products. These requirements have become tightly interdependent through long-term coevolution, so relocating an individual reaction can disrupt the balance of the network. Engineering strategies should therefore address the three classes of limitations described above. To address transport limitations, any proposed alteration in metabolite flux should be accompanied by a comprehensive assessment of the substrate specificity and transport capacity of the relevant transporters, rather than an assumption that endogenous transporters can automatically accommodate increased flux. To address cofactor limitations, compartmental reorganization requires a concurrent assessment of whether energy supply and reducing equivalent regeneration can meet the demands of the redesigned pathway, rather than a focus solely on carbon skeleton flux. To address by-product limitations, designs that generate H_2_O_2_ in compartments other than its native site of production should demonstrate, through stoichiometric analysis and in situ measurements, that H_2_O_2_-scavenging capacity is sufficient to accommodate the maximum anticipated rate of H_2_O_2_ production. Catalase, ascorbate peroxidase, and peroxiredoxins should not be regarded as interchangeable modules. Thus, compartmental reorganization is feasible in principle, but it requires an integrated design that accounts for all four requirements.

#### 2.2.4. Empirical Validation

(1) Natural evolutionary examples: C_2_ and C_4_ lineages demonstrate that the distribution of photorespiratory reactions among cell types can be reconfigured, often alongside coordinated changes in vein density, organelle abundance, metabolite transport, and cell-type-specific gene expression [76,77]. These examples support the feasibility of compartmental reorganization while highlighting the need for an integrated design. (2) Engineering examples: Targeting glycolate oxidase (GO) to chloroplasts increased photorespiration-dependent oxidative stress through sustained H_2_O_2_ production [27]. By contrast, co-expression of chloroplast-targeted GO, malate synthase (MS), and catalase (CAT) increased dry biomass and photosynthetic rates in engineered lines, supporting the potential of complementary H_2_O_2_-scavenging systems to mitigate oxidative stress [78]. Replacing GO with bacterial glycolate dehydrogenase avoided the direct production of H_2_O_2_ during glycolate oxidation [11]. The inclusion of CAT in the engineered glycolate oxidation cycle (GOC) in potato [14] is consistent with the need to manage H_2_O_2_ production, although it does not establish that chloroplast redox risks were fully eliminated. Collectively, these examples highlight the importance of coordinating metabolite transport, cofactor supply and demand, and by-product removal to sustain flux through the redesigned pathway. Two additional pathways provide further insights. The FD2-type GMA bypass couples chloroplast glycolate oxidation with subsequent complete decarboxylation, while co-expressed ascorbate peroxidase (APX) scavenges the H_2_O_2_ generated during glycolate oxidation. This design improved photosynthesis, biomass, and yield without compromising the seed-setting rate in rice [15], suggesting that appropriate auxiliary components can help manage H_2_O_2_ production. The ND1-type BHAC bypass operates in the cytosol and conserves nitrogen while promoting the accumulation of four-carbon metabolites in *Arabidopsis* [16]. This example shows that engineered bypasses need not be confined to chloroplasts, although intercompartmental transport, cofactor balance, and redox homeostasis must be evaluated for each design.

#### 2.2.5. Assessment of Evidence Completeness

The engineering studies discussed above have primarily addressed by-product management, with both successful and unsuccessful outcomes highlighting the need to coordinate H_2_O_2_ production with scavenging capacity. However, these studies provide limited evidence for transporter compatibility and lack quantitative validation of the balance between cofactor supply and demand.

### 2.3. Physiological–Ecological Constraints

#### 2.3.1. Evolutionary Origin

The photorespiratory network is a product of long-term evolution and is tightly integrated with cellular physiology and metabolism. Cyanobacteria require the metabolic removal of 2-phosphoglycolate (2-PG) generated by Rubisco oxygenase activity to sustain autotrophic growth under ambient air conditions [3]. Following plastid endosymbiosis, pathways for 2-PG metabolism became integrated into the eukaryotic host. Over evolutionary time, photorespiration became closely coupled to cellular redox homeostasis, carbon, nitrogen, and sulfur metabolism, and source–sink relations, acquiring functions beyond carbon salvage and becoming a metabolic hub. The canonical three-compartment organization, spanning chloroplasts, peroxisomes, and mitochondria and involving peroxisomal glycolate oxidase (GO), became established during the evolution of the green plant lineage [22]. This organization is broadly conserved across land plants, from liverworts to angiosperms, and photorespiration can serve as an electron sink that helps balance photosynthetic energy metabolism [67]. Its evolution has also been closely linked to that of photosynthetic carbon-concentrating mechanisms [22]. However, the establishment of this three-compartment organization did not mark the end of photorespiratory evolution. In C_2_ plants, often described as C_3_–C_4_ intermediates, glycine decarboxylase complex (GDC) activity is largely restricted to bundle sheath cells. Glycine produced in mesophyll cells is transported to bundle sheath cells for decarboxylation, creating a photorespiratory CO_2_-concentrating mechanism that can represent an intermediate stage in the evolution of C_4_ photosynthesis [76,77,79]. C_2_ photosynthesis has evolved independently in multiple lineages, often alongside changes in bundle sheath development, vein density, and organelle distribution [76,80]. In some lineages, further anatomical and biochemical modifications gave rise to C_4_ photosynthesis [76]. The C_4_ pathway concentrates CO_2_ around Rubisco, thereby suppressing its oxygenase activity and reducing photorespiration, particularly under conditions that favor Rubisco oxygenation.

#### 2.3.2. Formation and Nature of Physiological–Ecological Flexible Constraints

Over evolutionary time, photorespiration has become integrated with diverse physiological and ecological processes across multiple spatial and temporal scales. (1) Seconds to minutes: photorespiration provides an electron sink that contributes to energy and redox balance under high-light conditions. Comparisons across representative land plant groups have revealed differences in photorespiratory electron sink capacity, with higher capacities reported in the order liverworts, ferns, gymnosperms, and angiosperms; in the gymnosperms examined, photorespiration was identified as the predominant electron sink under the conditions tested [67]. These findings suggest that the contribution of photorespiration to electron dissipation varies among land plant lineages. Its electron sink function primarily reflects the consumption of ATP and reducing equivalents associated with photorespiratory metabolism, rather than H_2_O_2_ signaling itself. (2) Minutes to hours: photorespiration is closely coupled to nitrogen, sulfur, and one-carbon metabolism through reactions distributed across chloroplasts, peroxisomes, and mitochondria. Ammonia released by the glycine decarboxylase complex (GDC) is reassimilated through the chloroplast glutamine synthetase 2 (GS2)/ferredoxin-dependent glutamate synthase (Fd-GOGAT) system, sustaining a high-flux internal nitrogen cycle. In *Arabidopsis* mutants with impaired GDC activity, the expression of genes involved in sulfur reduction and assimilation is altered [30], while isotope-tracing experiments have shown that photorespiratory metabolism stimulates sulfur assimilation [81]. The coupled activities of GDC and serine hydroxymethyltransferase (SHMT) link photorespiration to folate-mediated one-carbon metabolism, potentially influencing nucleotide synthesis and methyl donor availability, including the supply of S-adenosylmethionine (SAM), through connections with folate metabolism and the methionine cycle [82,83,84]. Thus, photorespiration functions not merely as a carbon salvage pathway associated with CO_2_ loss, but also as a metabolic hub linking carbon, nitrogen, and sulfur metabolism with cellular methylation potential. (3) Hours to daily and seasonal scales: photorespiration is linked to source–sink relations, development, and stress responses at the cellular, tissue, and whole-plant levels. At the cellular level, the identification of the vacuolar glycerate transporter NITRATE TRANSPORTER 1/PEPTIDE TRANSPORTER FAMILY 8.4 (NPF8.4) revealed a mechanism by which glycerate, a photorespiratory carbon intermediate, can be sequestered in vacuoles under nitrogen-deficient conditions, contributing to the regulation of carbon–nitrogen balance [68]. At the tissue level, perturbation of plastidic 2-phosphoglycolate metabolism results in starch and H_2_O_2_ accumulation in guard cells and alters stomatal development and movement, linking photorespiratory metabolism to stomatal function [85]. These findings suggest that changes affect not only carbon assimilation but also stomatal conductance and water-use efficiency, potentially through associated metabolic and signaling processes. At the whole-plant level, increased leaf net carbon assimilation is the first step toward higher yield: additional assimilates must be loaded into the phloem, transported, and taken up and utilized by developing grains, tubers, storage roots, or fruits. The number of sink organs, their potential storage capacity and nitrogen demand, and the duration of assimilate accumulation collectively influence the allocation of newly assimilated carbon [17].

#### 2.3.3. Engineering Implications

Photorespiratory engineering strategies should not be evaluated solely on the basis of short-term changes in net photosynthesis or biomass; their effects should also be assessed at three levels. (1) Redox and energy balance: For designs that substantially reduce Rubisco oxygenation or decrease photosynthetic electron consumption, measurements should include Photosystem I (PSI) acceptor-side limitation under fluctuating light, P700 redox dynamics, non-photochemical quenching (NPQ) dynamics, and ROS accumulation. For bypasses that modify only downstream 2-PG metabolism, the effects on ATP and NAD(P)H balances and electron demand should first be quantified, rather than assuming that the electron-sink function is entirely lost. If a design reduces the electron-sink capacity of photorespiration, studies should determine whether alternative electron sinks can compensate. (2) C–N–S metabolism and methylation: Plant material with directly impaired GDC activity should be evaluated for effects on nitrogen recycling, sulfur metabolism, and one-carbon metabolism. A bypass that does not directly affect GDC should not be assumed to weaken nitrogen recycling without supporting evidence. The effects of photorespiration suppression on nitrate assimilation depend on the species and nitrogen source and should not be generalized. For designs that substantially redirect glycine, serine, or formate flux, potential effects on methylation and defense metabolism should be treated as testable hypotheses. (3) Source–sink coordination and stomatal function: Source–sink coordination should be considered alongside leaf photosynthesis at the design stage, rather than addressed only after field trials reveal limitations. Increased leaf carbon assimilation will translate into higher yield only if assimilates can be transported to and used by sinks with sufficient capacity. Designs should also be evaluated for their effects on stomatal conductance and water-use efficiency. Because physiological and ecological constraints are context-dependent, the feasible engineering window will vary with conditions. Targets with weaker network coupling, or systems whose buffering capacity can be strengthened in advance through breeding or agronomic interventions, may offer viable routes to redirect photorespiratory flux while preserving existing physiological functions. The goal should not be to minimize photorespiratory flux indiscriminately, but to selectively reduce its avoidable costs within physiological limits while preserving essential functions or providing compensatory mechanisms.

#### 2.3.4. Empirical Validation

The engineering implications discussed above are supported by evidence to varying degrees. (1) Redox and energy balance: Studies in tomato have shown that high-light-acclimated plants with strong photorespiratory capacity can use photorespiration as an additional electron sink following abrupt increases in light intensity, thereby limiting PSI over-reduction and photoinhibition [86]. This finding supports a role for photorespiration in redox buffering under fluctuating light and highlights the need to assess whether alternative electron sinks can compensate when photorespiratory electron-sink capacity is reduced. (2) Source–sink coordination: The decline in seed-setting rate was more pronounced in rice lines expressing the glycolate:glyoxylate aminotransferase (GCGT) bypass in genetic backgrounds with perturbed one-carbon (C_1_) metabolism, and under high-CO_2_ conditions; its amelioration by the subsequent rice sink-strength (RSS) module illustrates that yield benefits from increased leaf carbon assimilation depend on assimilate transport and sink capacity [12,13,87]. This evidence supports evaluating source–sink coordination at the design stage. (3) C–N–S metabolism and methylation: Recent studies have shown that photorespiration-derived formate can enter the cytosolic folate cycle and influence DNA methylation patterns. These effects were more pronounced in genetic backgrounds with perturbed one-carbon (C_1_) metabolism and under high-CO_2_ conditions [88]. This finding provides a mechanistic rationale for including C_1_ metabolism and methylation status in engineering assessments, although the magnitude of these effects and their agronomic consequences remain to be quantified systematically. Notably, the FD2-type GMA bypass increased rice yield without compromising seed-setting rate [15], in contrast to the decline observed in GCGT bypass lines. Although both are chloroplast-targeted bypasses, their different effects suggest that bypass design—including carbon fate and the site of decarboxylation—may influence source–sink outcomes. These contrasting results underscore the need to evaluate the physiological and ecological consequences of each design rather than infer them from subcellular localization alone.

#### 2.3.5. Assessment of Evidence Completeness

(1) Redox and energy balance: The study in tomato provides direct empirical evidence that photorespiration can serve as an electron sink and contribute to redox buffering under fluctuating light. (2) Source–sink coordination: Studies of the rice GCGT bypass and the RSS module together link leaf-level gains to agronomic outcomes by identifying source–sink limitations associated with the bypass and showing that enhanced sink capacity can alleviate these limitations. These findings support design-specific evaluation of source–sink coordination rather than the assumption that improved leaf photosynthesis will necessarily translate into increased yield. (3) C_1_ metabolism and methylation: Current evidence linking photorespiration-derived formate to DNA methylation highlights a potential mechanistic risk but does not establish that perturbations in C_1_ metabolism necessarily impair agronomic performance. Accordingly, for designs that substantially redirect glycine, serine, or formate flux, potential effects on C_1_ metabolism and methylation should be treated as testable risks rather than assumed outcomes.

### 2.4. Nested Relationships Among the Three Classes of Constraints and Engineering Implications

**Figure 2:**
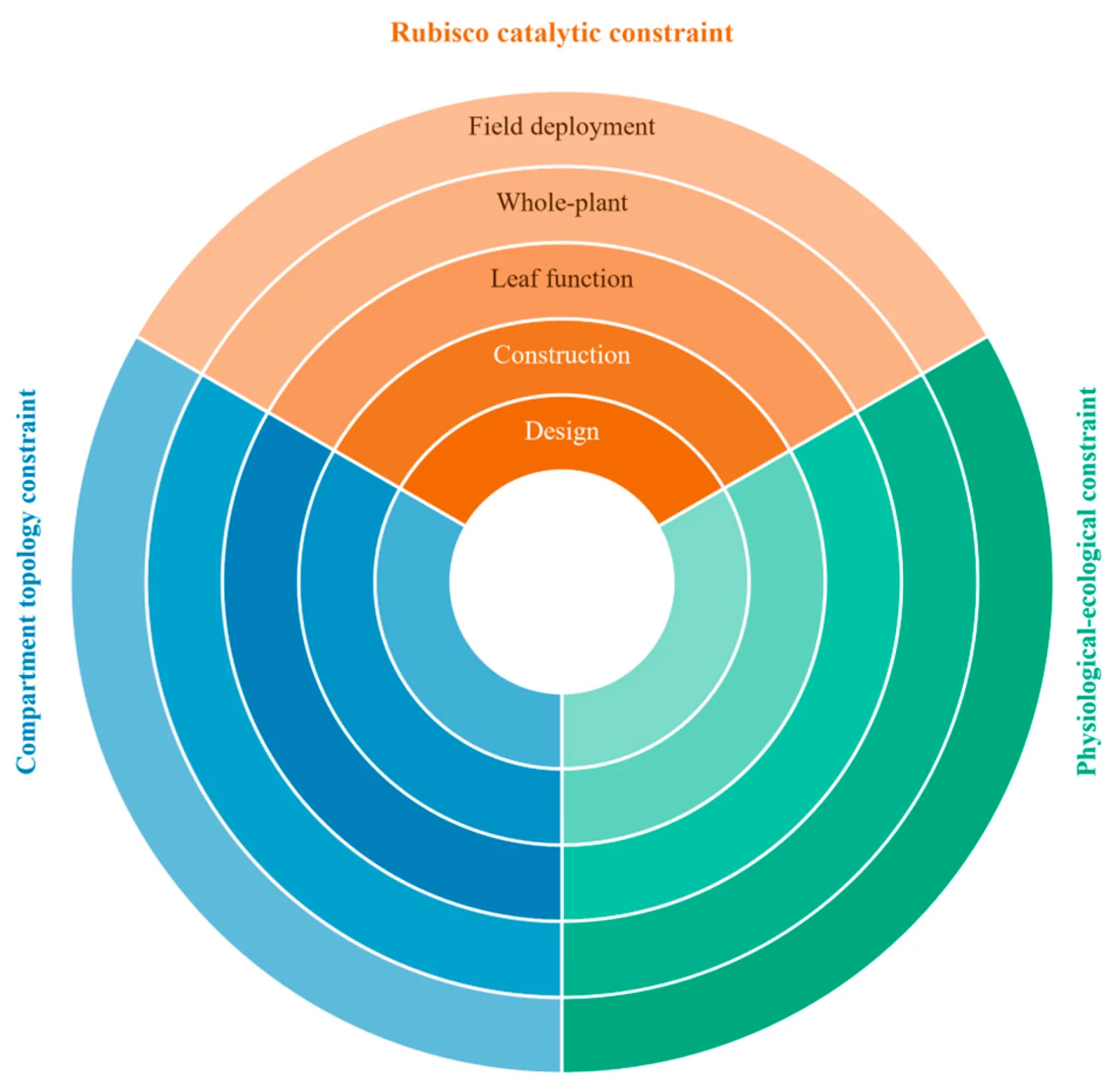
Relative influence of three classes of constraints across stages of photorespiratory engineering. Concentric rings represent successive engineering stages, from design (innermost ring) to field deployment (outermost ring). The three sectors represent Rubisco catalytic constraints (orange), compartmental topology constraints (blue), and physiological–ecological constraints (green). Within each ring, darker shading indicates a greater relative influence of the corresponding constraint class at that stage.

The three classes of constraints form a nested hierarchy (Figure 2). Catalytic constraints set the upper limit on 2-PG flux, and Rubisco engineering, particularly complete replacement of the enzyme, directly determines the magnitude of the flux entering downstream metabolism. Compartmental topology constraints limit the scope for reorganization at a given flux, requiring compartmental configurations to ensure transport closure, cofactor balance, and byproduct disposal. Physiological–ecological constraints form the outermost layer: even when compartmental closure is achieved, homeostasis at the whole-plant level and under varying environmental conditions, as well as the translation of metabolic gains into yield gains, remain subject to constraints imposed by redox balance, C–N–S coupling, source–sink coordination, and stomatal behavior. This nested hierarchy suggests that evaluation should proceed in reverse order: first, determine whether sink organs can accommodate gains achieved at the leaf level; next, assess whether compartmental configurations achieve closure; and finally, determine whether Rubisco specificity needs to be optimized concurrently. Proceeding in the forward direction risks adopting an “engineer Rubisco first, then observe the phenotype” approach—a form of local optimization. In contrast, reverse evaluation requires identifying, before engineering begins, which constraints will be activated at each level and which complementary components will be needed. Overcoming constraints at one level while neglecting those at others often leads to systemic imbalance. The conditions under which these constraints become operative also differ: catalytic constraints are activated by any change in kinetic parameters, regardless of its magnitude, whereas the activation of compartmental topology and physiological–ecological constraints depend on whether specific closure relationships are disrupted, with greater disruption requiring more compensatory components.

## 3. Constraint-Aligned Photorespiratory Engineering: Design Principles, Evaluation Framework, Validation Pipeline, and Applications

Photorespiratory engineering strategies vary substantially across studies, making it difficult to compare findings and draw generalizable conclusions. Existing general-purpose frameworks are not directly applicable: generic reporting frameworks do not adequately address photorespiration-specific features, such as flux partitioning, compartmental closure, C–N–S coupling, and the translation of leaf-level gains into sink growth or yield gains. Moreover, the standard Farquhar–von Caemmerer–Berry (FvCB) model [89] describes photorespiratory CO_2_ release within the conventional photorespiratory pathway and accounts for non-photorespiratory respiration in the light as a separate term. Applying this model without modification to plants with engineered bypasses may introduce systematic biases because these bypasses can alter both the location and the amount of CO_2_ released [90]. A dedicated framework for evaluation and validation is therefore needed. This review translates insights from evolutionary analysis into a practical framework for evaluating photorespiratory engineering, organized into four sections: design principles, a reporting framework, a validation pipeline, and case applications. The framework provides a checklist for reporting, a tool for assessing the completeness of evidence, and a basis for classifying conclusions as supported, unsupported, or indeterminate.

### 3.1. Design Principles

Five design principles are presented in Box 1.

Box 1 **Five design principles for photorespiratory engineering.**

Principle 1: Optimize within the feasible design space rather than maximize a single parameter. Increasing Rubisco CO_2_/O_2_ specificity (S_C/O) or reducing nominal CO_2_ release does not necessarily improve whole-plant efficiency. Designs must account for enzyme abundance, catalytic turnover, substrate affinity, the chloroplast CO_2_ environment, energy requirements, and CO_2_ refixation.

Principle 2: Ectopic reactions require evidence of compartmental closure. Relocated reactions require evidence of adequate substrate supply, product export, cofactor regeneration, and byproduct disposal. The consequences of H_2_O_2_ or CO_2_ production in chloroplasts cannot be assessed solely on the basis of transgene expression levels and final biomass.

Principle 3: Preserve or compensate for native functions rather than simply maintain native flux. Key questions are: Which functions are compromised? Are alternative modules available to fulfill these functions? Do these alternatives function effectively under relevant environmental conditions and at relevant developmental stages?

Principle 4: Match engineering complexity to experimentally identified bottlenecks. Multigene engineering is not inherently superior to simpler approaches. Compensatory modules should be added only when experimental evidence demonstrates that they are necessary.

Principle 5: Scale the depth of validation to the degree of deviation from the native network. Designs that alter Rubisco, suppress glycine decarboxylase (GDC) activity, generate reactive byproducts, or alter transgene expression in reproductive tissues require more extensive validation. Even lower-risk designs require validation using at least three independent transformation events and confirmation of heritable trait stability in the T_3_ generation or later.

When design principles conflict, no single principle takes precedence a priori; instead, the design is evaluated within the G×E×D×M×S context (see Box 2) to identify which constraints become limiting and whether the associated costs are acceptable.

Box 2 **G×E×D×M×S context matrix and interactions.**

G—Genetic background: Species, cultivar, ploidy, leaf anatomy, Rubisco kinetics, baseline photorespiration, nitrogen utilization, and transformation event. Source–sink traits are assigned to S. Validate designs using multiple independent transformation events; for deployment, test them in multiple cultivar backgrounds.

E—Environment: Light intensity and fluctuations, temperature, CO_2_ concentration, water availability, nitrogen and sulfur supply, and combined stresses. Report the specific treatments, developmental stages assessed, and effect sizes. Include CO_2_ scenarios relevant to the intended deployment period (e.g., 2050 and 2100; [91,92]).

D—Engineering design: Bypass type, reaction chemistry, subcellular localization, promoter, targeting peptide, regulation of transport, suppression of the native pathway, and compensatory modules.

M—Management: Sowing date, planting density, fertilization, irrigation, canopy management, and pest control. Evaluate M jointly with D and E. Trials across seasons and years provide environmental replication and strengthen the evidence base; seasons and years are not management variables.

S—Source–sink status and developmental stage: Vegetative and reproductive stages; sink organ type, number, capacity, and timing of establishment; nitrogen demand; and grain filling. Assess whether the effects of a design reverse across developmental stages.

Interactions: Assessment of E×D interactions under different CO_2_ scenarios and M×E interactions involving water management is mandatory for all designs. Assessment of G×D interactions is required when applicability across cultivars is claimed, and assessment of D×S interactions is required when transgene expression extends into reproductive development. Assessment of other interactions is optional; any untested interactions should be identified in the Discussion.

Representative environments: Early-stage studies conducted in greenhouses or under controlled conditions [93] need not include sites subject to major environmental limitations, but they must describe how the experimental conditions differ from those in the target region. Late-stage studies must cover gradients in the major limiting factors, including at least one water- or temperature-limited site and one site not limited by the selected factor. Regionally relevant soil stresses must also be included where present. Site selection should proceed by ranking the limiting factors, selecting gradients for the two highest-ranked factors, and including at least one site exposed to combined stresses. Sites exposed to combined stresses are mandatory when stress adaptation is claimed.

Core metadata: Genotype, temperature, light, CO_2_ concentration, water availability, nitrogen supply, engineering design, and developmental stage. Additional module-specific requirements are provided in Supplementary Tables S1 and S2. Report environmental treatments, developmental stages, and effect sizes, as detailed under the environmental dimension (E). G×E×D interactions may be nonlinear, and their effects may reverse direction across contexts; report the evidence tier, species, experimental conditions, and associated uncertainties.

### 3.2. The Eight-Dimensional Reporting Framework

The eight dimensions provide a disclosure tool for identifying reporting gaps and comprise a minimum set of indicators derived from the three classes of constraints (Table 1). Reporting is organized into four modules: core (covering all eight dimensions), risk-dependent (Supplementary Table S1), claim-specific (Supplementary Table S2), and region-specific (covering the same elements, with specifications adapted to the target region). Omission of any required indicator constitutes a reporting gap; otherwise, optional indicators become mandatory when the corresponding module applies. The acceptance criteria are defined by the evidence tiers in the validation pipeline.

The eight dimensions cover the following: (1) integrity of engineering implementation (a prerequisite); (2) flux and carbon fate (compartmental topology constraints); (3) catalysis and the chloroplast gas environment (Rubisco catalytic constraints); (4) energy–redox closure (compartmental topology constraints); (5) C–N–S–C_1_ metabolic coupling (physiological–ecological constraints); (6) source–sink and developmental coordination (physiological–ecological constraints); (7) agronomic performance and product quality (physiological–ecological constraints); and (8) genetic stability, environmental robustness, and reproducibility (cross-cutting). A single dimension may address multiple classes of constraints, and a single class of constraints may require assessment across multiple dimensions.

### 3.3. G×E×D×M×S Context Matrix

The effectiveness of photorespiratory engineering depends on genetic background (G), environment (E), engineering design (D), management (M), and source–sink status and developmental stage (S). The G×E×D×M×S matrix (Box 2) does not require testing every possible combination of these factors. Rather, its application requires clearly defining the intended deployment context, selecting representative environments, and reporting core metadata.

### 3.4. Five-Tier Hypothesis-Driven Validation Pipeline

The validation pipeline specifies the sequence of measurements and the criteria for evaluating outcomes. The overarching hypothesis is that a given photorespiratory engineering design can deliver agronomic gains attributable to the intended mechanism within a predefined G×E×D×M×S context, while maintaining key metabolic, reproductive, and quality indicators within prespecified non-inferiority margins.

This overarching hypothesis is divided into five sequential validation tiers (H1–H5), organized under two sub-hypotheses. Sub-hypothesis A concerns whether the design has been successfully implemented and functions as intended in plants and encompasses H1 (engineering implementation) and H2 (mechanism and flux closure). Sub-hypothesis B concerns whether the design delivers reproducible agronomic gains and encompasses H3 (dynamic physiological robustness), H4 (whole-plant and agronomic translation), and H5 (deployment stability). Each tier is assigned one of three outcomes: supported (tested, with results consistent with the hypothesis), not supported (tested, with results inconsistent with the hypothesis, requiring design modification or restriction of the associated claim), or indeterminate (not tested, not assessable using the available methods, or supported by insufficient evidence). “Not evaluated” denotes a procedural status rather than a fourth evidentiary outcome: it indicates that evaluation of a downstream tier was halted because a prerequisite hypothesis was not supported. Distinguishing between “not supported” and “indeterminate” is particularly important. For example, if bypass flux partitioning has not been measured directly, H2 is classified as indeterminate, indicating the need for additional measurements. Classifying it as not supported would incorrectly imply that experimental evidence contradicts the proposed flux-based mechanism of the gains and would therefore misdirect subsequent decisions toward design modification rather than further measurement.

States propagate downstream through the validation pipeline according to three types of rules. First, attribution rules govern the strength of mechanistic attribution: if H1 is indeterminate, downstream evaluation continues, but mechanistic attribution is withheld, and the evidence gap is designated as an “H1-type gap”; if H2 is indeterminate, downstream mechanistic attribution is limited to qualitative interpretation. Second, termination rules determine whether downstream evaluation proceeds: if H1 is not supported, H2–H5 are designated as “not evaluated”; if H3 is not supported because of irreversible damage or premature senescence, H4 and H5 are designated as “not evaluated.” Any modification to the design requires re-evaluation starting from H1; the addition of supplementary data alone does not constitute a design modification. Third, claim-restriction rules limit the scope of downstream claims without changing the evaluation states assigned to downstream tiers. For example, if H3 is indeterminate, H4 is still evaluated, but claims of dynamic physiological robustness cannot be made; if H4 is not supported or is indeterminate, the scope of deployment-related conclusions at H5 is restricted.

The specific hypotheses and criteria for each tier are presented in Table 2. Each tier is assigned one of three outcomes—supported, not supported, or indeterminate—according to predefined criteria and thresholds. For example, H1 requires validation using at least three independent transformation events and demonstration of transgene stability through at least the T_3_ generation to account for insertion-site effects and assess stability across generations. These requirements are proposed here as operational criteria, informed by the emphasis on molecular characterization and genetic stability in transgenic crop safety assessment frameworks (e.g., EFSA, 2011). H2 requires quantitative flux partitioning using ^13^C or ^14^C tracers to determine the flux diverted through the bypass or the residual flux through the native pathway [94,95]. For complete Rubisco replacement strategies, which do not involve an engineered bypass, the H2 evaluation focuses on verifying the new carbon flux steady-state. This requires quantifying the altered CO_2_ release ratio or photorespiratory flux using isotopic tracing or extended gas-exchange models that couple the introduced enzyme’s kinetics with the chloroplast environment. Under this framework, semi-quantitative evidence alone is insufficient: reliance solely on indirect inference is recorded as a reporting gap in Dimension 2, and H2 is classified as indeterminate. This requirement is intended to guide future validation rather than invalidate existing findings: without adequate flux quantification, attribution of the observed gains to the proposed flux-based mechanism remains provisional.

The combination of evaluation states across tiers determines the permissible scope of claims. Representative combinations are shown in Table 3. For combinations not listed, claims are permissible only if they satisfy the restrictions imposed by all applicable tiers. A tier classified as not supported precludes claims that require support from that tier, including downstream claims that depend on it, whereas a tier classified as indeterminate precludes extrapolations that require the evidence missing from that tier. Before experiments begin, the primary endpoints, tier-specific thresholds, non-inferiority margins, target deployment context, and the proportion defining a “majority” at H5 must be prespecified and documented. These specifications must be submitted with the study report. Post hoc changes to these specifications invalidate the corresponding tier evaluations under.

Case applications: Five published cases were evaluated sequentially at the element, context, and validation-pipeline levels (Table 4). These cases were selected specifically to assess the scope and completeness of the proposed framework and identify its limitations. They encompass four engineering strategies: complete decarboxylation in chloroplasts (FD2/GMA) [15], partial decarboxylation in chloroplasts (PD1/Kebeish [11] and PD2/GCGT [13]), a cytosolic bypass (ND1/BHAC) [16], and complete Rubisco replacement [8]. The cases span three levels of experimental validation—greenhouse trials, single-location field trials, and multi-location field trials—and include both positive and negative outcomes. These five cases are not representative of the field as a whole, and the gaps identified should not be assumed to occur across all photorespiratory engineering studies.

Element layer: All five cases have reporting gaps. Flux partitioning (Dimension 2) was not quantified in any of the cases, and quality indicators (Dimension 7) were not reported for the rice cases. The Rubisco replacement case triggers the risk-dependent module, which requires a complete set of kinetic parameters, Γ*, and estimates of chloroplast CO_2_ concentration; however, these requirements were not fully met. FD2 and PD2 trigger the claim-matching module, requiring additional reporting on sink capacity, transport, seed-setting rate, and product quality.

Context layer: Coverage of the G×E×D×M×S context is incomplete in all five cases. Because of the low CO_2_ affinity of HnRubisco, the Rubisco replacement case was evaluated under.

1% CO_2_, a controlled condition that does not represent the intended deployment context. PD2 identified a transport bottleneck, whereas FD2 reported evidence of source–sink balance, providing a contrasting case.

Pipeline layer: H2 is classified as indeterminate in all five cases because *in planta* flux partitioning was not quantified. For PD2 (GCGT), the significant reduction in seed-setting rate, despite enhanced vegetative growth, leads to an H4 classification of not supported. In contrast, FD2 (GMA) maintained yield; however, because quality indicators were not reported, its H4 classification remains indeterminate. H3 remains indeterminate in all five cases because responses to dynamic stress conditions were not tested, and H5 is classified as either indeterminate or not evaluated, depending on the case. All five cases could be assessed using the three-layer sequence, demonstrating the framework’s practical applicability. The resulting recommendations fall into two categories: additional evidence collection and design or context adjustment. Additional evidence is needed on flux partitioning for all bypass cases, product quality for FD2 and PD2, performance in crop genetic backgrounds for PD1, the need for source–sink compensation in PD2, and agronomic efficacy and source–sink coordination for ND1. Design or context adjustments include evaluating carbon-concentrating mechanisms (CCMs) or elevated-CO_2_ deployment scenarios for Rubisco replacement. Any design modification requires re-evaluation starting from H1, with downstream judgments subject to the applicable propagation rules. For example, H4 for GCGT+RSS would be classified as indeterminate rather than not supported pending evaluation of the modified design.

Limitations: This retrospective analysis demonstrates that the framework can be applied to published cases but does not establish its predictive validity. Assessments were conducted by the authors without testing inter-rater reliability. Moreover, reliance on published studies may introduce publication bias by underrepresenting unsuccessful designs. These limitations motivate three priorities for future work: conducting independent assessments by multiple evaluators and reporting inter-rater agreement; tracking changes in evaluation states following additional measurements; and prospectively testing predictive validity using a preregistered study design.

## 4. Conclusions

### 4.1. Core Insights from an Evolutionary Perspective

The evolutionary history of photorespiration reflects both the emergence of constraints and opportunities for metabolic reconfiguration. Oxygenase activity is an intrinsic property of Rubisco, making 2-PG removal necessary whenever this reaction occurs. In land plants, the need to metabolize 2-PG has helped integrate chloroplasts, peroxisomes, and mitochondria into a tightly coupled network linked to redox buffering, C–N–S and one-carbon metabolism, source–sink coordination, development, and stress responses. These interdependencies have shaped three classes of constraints—Rubisco catalytic, compartmental topology, and physiological–ecological constraints—that represent context-dependent limits rather than fixed barriers. The repeated emergence of C_2_ and C_4_ photosynthesis illustrates the potential for reorganizing photosynthetic and photorespiratory metabolism, although such changes require coordinated modifications to anatomy, transport, and regulation over evolutionary timescales. Engineering interventions, by contrast, seek to achieve functional integration within one or a few generations. This contrast underscores the need to translate evolutionary insights into quantifiable design limits and explicit criteria for evaluating evidence.

### 4.2. Framework Positioning and Boundaries

The eight assessment dimensions, G×E×D×M×S context matrix, and H1–H5 validation pipeline presented in this review together form a minimum evidence framework aligned with the three classes of constraints. The framework serves three functions: (1) a reporting checklist comprising the eight dimensions and the G×E×D×M×S context matrix, specifying the information that must be reported; (2) a tool for assessing evidence completeness through H1–H5 state assignments, determining whether the available evidence is sufficient to address specific scientific questions; and (3) a reference for evaluating outcomes and defining the permissible scope of claims, distinguishing among three distinct states—supported, not supported, and indeterminate. The framework explicitly defines what constitutes sufficient evidence and what constitutes an acceptable outcome. However, it does not rank engineering strategies, assign composite scores, or replace researchers’ scientific judgment.

The framework’s central function is to distinguish insufficient evidence from evidence against a hypothesis: the former calls for additional measurements or other evidence, whereas the latter may warrant design modification or restriction of the associated claim. Retrospective application to five published cases illustrates the relevance of this distinction to current research. The absence of quantitative flux partitioning is a common gap in mechanistic attribution across these cases, leading to an H2 classification of indeterminate and indicating the need for additional evidence rather than establishing design failure. Negative findings must also be interpreted within the scope of the tier being evaluated. The contrasting H4 classifications of the GMA bypass case and rice GCGT—supported and not supported, respectively—do not establish the superiority of one design over the other; rather, they highlight the importance of evaluating source–sink coordination when assessing whether physiological gains translate into yield gains, particularly in crops harvested for seeds. Similarly, the H3 classification of not supported for rice GDCH knockdown [96] does not rule out GDC regulation as an engineering strategy; instead, it identifies a physiological limitation under the genetic background and environmental conditions tested. The scope of the framework must also be made explicit. It addresses the biological evaluation of photorespiratory engineering, not regulatory approval, economic viability, or farmer adoption. Its state-propagation logic could, in principle, be adapted to other engineering strategies, such as the introduction of carbon-concentrating mechanisms (CCMs) or the conversion of C_3_ crops to C_4_ photosynthesis, but the tier-specific criteria would need to be redefined and validated for each application.

### 4.3. Concluding Remarks

The ultimate criterion for success in photorespiratory engineering is to achieve agronomic gains that are attributable to the intended mechanism, reproducible across years and locations within the target deployment context, and accompanied by no unacceptable deterioration in key traits, as defined by prespecified non-inferiority margins. The framework presented here provides a practical structure for evaluating the evidence needed to establish such success. Its value lies in enabling findings from studies with positive or negative outcomes to be compared and integrated within a common evaluative framework.

Evolutionary history reveals that the constraints on photorespiratory engineering are not necessarily insurmountable barriers but context-dependent limits. The framework translates these constraints into measurable indicators and explicit criteria for evaluating evidence. Rather than asking only whether a pathway can be engineered, future research should determine which genetic backgrounds, environmental conditions, and developmental stages permit successful implementation, which complementary modifications are needed, and what evidence is sufficient to demonstrate success. When a design cannot sustain autotrophic growth at current atmospheric CO_2_ concentrations, as in the Rubisco replacement case [8], the framework does not treat this result as evidence that Rubisco replacement is universally infeasible. Instead, it identifies a mismatch between the kinetic properties of the introduced Rubisco and the chloroplast CO_2_ environment, motivating tests of complementary approaches such as carbon-concentrating mechanisms (CCMs), alternative Rubisco variants, or deployment conditions with higher CO_2_ availability. Similarly, when gains observed under controlled conditions fail to translate into reproductive performance in the field, as illustrated by the reduced seed-setting rate in the PD2/GCGT case [13], the framework does not dismiss the bypass as universally ineffective. Rather, it directs attention to potential source–sink limitations and the evidence needed to justify compensatory modules. This shift toward context-specific diagnosis and evidence-based redesign is important for translating photorespiratory engineering from experimental research into agricultural applications. The framework remains open to revision: as flux measurements become more widely accessible and multi-environment data accumulate, the eight assessment dimensions can be refined and, where appropriate, streamlined, H1–H5 thresholds recalibrated, and the assessment of G×E×D×M×S interactions improved. Its core logic—translating evolutionary constraints into explicit evidence requirements and distinguishing evidence gaps from evidence against a hypothesis—nevertheless provides a common language for the field. By enabling comparisons across laboratories, crops, and environments, the framework can help photorespiratory engineering progress from the accumulation of individual case studies toward systematic optimization.

## 5. Future Directions

### 5.1. Quantitative Flux Partitioning as a Standard Practice

Without quantitative data on flux partitioning between engineered bypasses and native pathways, attribution of observed gains to the proposed mechanism remains provisional. Quantitative flux analysis using ^13^C or ^14^C tracers should become a core component, rather than an optional measurement, of photorespiratory engineering research. The development of standardized protocols and reporting guidelines will be essential for moving the field from phenotypic observation toward mechanistic attribution.

### 5.2. Early Evaluation of Compartmental Dynamics and Source–Sink Coordination

Changes in H_2_O_2_ levels, NADH/NADPH pools, and ATP availability in compartments hosting engineered reactions may affect both metabolism and signaling. Accordingly, the functional status of the photosynthetic apparatus under fluctuating light, stomatal conductance, and water-use efficiency should be incorporated into routine evaluations. Source–sink coordination should not be assessed only after poor field performance has been observed; it should also be considered during design and evaluated alongside leaf photosynthesis in early-stage experiments. The transport bottleneck identified in rice GCGT and the reported effects of RSS-mediated compensation highlight the importance of assessing source–sink limitations at an early stage.

### 5.3. Multi-Environment Validation and Iterative Refinement of the Framework

Rising atmospheric CO_2_ may reduce the relative benefits of photorespiratory engineering in some contexts [97,98]; therefore, results obtained at current CO_2_ concentrations should not be extrapolated directly to future scenarios. Replication across multiple locations, years, and cultivar backgrounds is essential for assessing the context dependence of these benefits. The framework itself also requires empirical validation. As additional cases become available, inter-rater reliability, predictive validity, and indicator redundancy should be assessed to refine and, where appropriate, streamline the framework. To reduce selective reporting when researchers evaluate their own studies, studies claiming suitability for field deployment should be preregistered, with primary endpoints, evaluation thresholds, and target deployment contexts specified before trial initiation.

## Figures and Tables

**Table 1:** Eight-dimensional elements.

Dimension	Corresponding Constraint	Question Addressed	Minimum Indicators	Optional Indicators (Examples)
1. Engineering implementation integrity	Prerequisite	Is the design realized as intended?	Construct and copy/editing status; subcellular localization; independent transformation events; key enzyme activities	Protein abundance; insertion site/copy number
2. Flux and carbon fate	Compartmental topology	Where does carbon actually flow?	Quantitative flux partitioning between bypass and native pathway; if not measurable, report reason and bounds	Intermediate pool sizes; ^13^C-labeling dynamics
3. Catalysis and chloroplast gas environment	Rubisco catalytic	Is the Rubisco performance combination feasible?	Rubisco content and activation state; A–Ci fitting parameters must report measurement model. Rubisco engineering additionally requires full kinetic parameters (k_cat_, K_C_, K_O_, S_C/O_), Γ*, chloroplast CO_2_ estimates	Γ* for bypass engineering; K_C_/K_O_ (mandatory when triggered)
4. Energy–redox closure	Compartmental topology	Are energy/reducing power budgets and byproduct disposal balanced?	ATP/NADPH budget based on actual flux; H_2_O_2_-producing designs must report scavenging capacity	NAD(P)H/NADP^+^ ratio; lipid peroxidation
5. C–N–S–C_1_ metabolic coupling	Physiological–ecological	Do carbon gains come at the cost of nutrients/C_1_?	Ammonia reassimilation flux or nitrogen recovery efficiency; glycine/serine ratio; major amino acid profiles	SAM/SAH; sulfur assimilation flux
6. Source–sink and developmental coordination	Physiological–ecological	Can leaf gains be transported and received?	Sink organ number and potential capacity; at least one transport/loading indicator	Harvest index; sugar loading/unloading rates
7. Agronomic performance and product quality	Physiological–ecological	Do gains have agricultural value?	Net photosynthesis and biomass; specified yield metrics and plot specifications; food crops reporting yield must include quality indicators	Yield components; processing/nutritional quality
8. Genetic, environmental, and replication stability	Cross-constraint	Are effects reproducible and deployable?	Independent events, generations, locations, years; effect sizes and variance; adverse phenotypes and ineffective events	G×E variance decomposition; heritability

Note: When an indicator cannot be measured, report the reason and provide bounds on its value or alternative evidence. Omission of any required indicator constitutes a disclosure gap. Otherwise, optional indicators become mandatory when the risk-dependent or claim-matching module applies (Supplementary Tables S1 and S2). For plants with engineered photorespiratory bypasses, A–C_i_ curve fitting must use extended models that explicitly incorporate bypass fluxes; alternatively, estimation methods that do not rely on standard FvCB assumptions may be used. The methodological basis must be reported in either case. For food crops, quality indicators are mandatory whenever the yield of harvested organs is reported.

**Table 2:** H1–H5 criteria and operational definitions.

Tier	Hypothesis	Supported	Not Supported	Indeterminate	Operational Definitions and Quantitative Standards
H1	Design realized as intended	Protein correctly localized and enzyme activity reaches design value	Expressed but localization or enzyme activity below design value, or target protein not detected	Localization or enzyme activity not reported	Independent transformation events ≥ 3; segregating line stability ≥ T_3_
H2	Bypass carries measurable flux; budget closure achieved	Flux partitioning quantified and gaps explainable	Quantified but gaps unexplainable	Only bounds or no estimates	Requires ^13^C/^14^C flux partitioning (steady-state or non-steady-state) to provide diversion rate or residual native flux
H3	Gains do not translate into damage under fluctuating conditions	Gains maintained and recovery capacity preserved	Irreversible damage or premature senescence	Not validated under target fluctuating conditions	Recovery metrics: PSI acceptor-side limitation, P700 oxidation, NPQ kinetics, ROS accumulation and scavenging rates; recovery time window set by target environment fluctuation cycles
H4	Leaf gains translate into yield and quality	Yield or quality improved and not below boundaries	Seed-setting rate or harvest index significantly declined, or quality exceeds boundaries	Does not meet standard cultivation metrics or quality not measured	Standard plot yield: ≥6 m^2^ (grain crops) or ≥10 m^2^ (tuber/root crops); randomized block design, ≥3 biological replicates; edge effects controlled; yield normalized per harvest area. Food crops must report quality.
H5	Gains reproducible across locations and years	Consistent direction in majority of locations and years	Inconsistent direction or systematic adverse phenotypes	Insufficient replicate locations or years	Majority: at least 2/3 of sites consistent direction and G×E interaction not significant (α = 0.05); if ≤3 sites, all must be consistent

Note: The framework explicitly defines what constitutes sufficient evidence and what constitutes an acceptable outcome, distinguishing insufficient evidence from evidence against a hypothesis.

**Table 3:** Representative H1–H5 state combinations and permissible claim scope.

H1–H5 States	Permissible Claims	Impermissible Claims
H1 supported + H2–H5 indeterminate	Preliminary positive phenotypes under tested conditions	Mechanistic attribution, agronomic efficacy, robustness, deployability
H1, H4 supported + H2, H3, H5 indeterminate	Evidence for agronomic efficacy (mechanism gap)	Mechanistic attribution, robustness, deployability
H1–H4 supported + H5 indeterminate	Candidate design for field deployment	Deployment stability, cross-varietal promotion
All supported	Evidence-supported feasible solution	Extrapolation to crops or environments outside the framework
H1 not supported + downstream all not evaluated	Reporting value limited to engineering failure	Any downstream conclusions
H1 supported + H3 not supported	H3-level negative findings reportable	Any agronomic efficacy statements
H1, H3 supported + H4 not supported	Local agronomic bottleneck identified	Yield increase statements, deployability

Note: For state combinations not listed, claims are permissible only if they satisfy the restrictions imposed by all applicable tier states. A tier classified as not supported precludes claims that require support from that tier, including downstream claims that depend on it; a tier classified as indeterminate precludes extrapolations that require the evidence missing from that tier. Tiers not explicitly listed in a given combination are handled according to the propagation rules described in Section 3.4.

**Table 4:** Comparative framework application to five cases.

Framework Element	FD2 (GMA Bypass)	PD1 (Kebeish Pathway)	PD2 (GCGT Bypass)	ND1 (BHAC Bypass)	Rubisco Replacement
Constraint triggered	Compartmental topology	Compartmental topology	Physiological–ecological	Compartmental topology	Rubisco catalytic
Eight-dimensional disclosure	1, 6 reported; 2–5, 7, 8 not reported	1, 3, 4 reported; 2, 5, 6, 8 not reported; 7 not required	1, 2, 6 reported; 3–5, 7–8 not reported	1, 5 reported; 2–4, 6–8 not reported	1, 3 reported; 2, 4–8 not reported; 7 not required
Risk/claim module applicability	H_2_O_2_ produced in non-native compartment; co-expressed ascorbate peroxidase (APX) scavenges it, addressing the risk module; claims agronomic value → Dimensions 6, 7 added	Risk module not applicable (design circumvents H_2_O_2_); claims agronomic value → Dimensions 6, 7 added	Suppresses chloroplast envelope transport → Dimension 2 extended requirements; claims agronomic value → Dimensions 6, 7 added	Risk module not applicable; no explicit agronomic claims	Directly alters Rubisco catalytic center → Dimension 3 extended requirements
G×E×D×M×S coverage	G single background; E greenhouse/field; S source–sink balance reported	G single background; E greenhouse; M/S not recorded	G single background; E greenhouse/field; S transport bottleneck identified	G single background; E greenhouse; M/S not recorded	G single background; E greenhouse + 1% CO_2_; M/S not recorded
H1–H5 states	H1 supported; H2 -H5 indeterminate	H1 supported; H2–H5 indeterminate	H1 supported; H2 indeterminate; H3 indeterminate; H4 not supported; H5 indeterminate	H1 supported; H2–H5 indeterminate	H1 supported; H2–H5 indeterminate
Permissible claim scope	Evidence for yield and biomass increase; mechanistic attribution not permissible	Evidence for greenhouse biomass increase; mechanistic attribution not permissible	Seed-setting rate declined, source–sink bottleneck identified; mechanistic attribution not permissible	Evidence for nitrogen retention and C_4_ metabolite accumulation; agronomic efficacy not permissible	Mechanism closure (controlled, high-CO_2_ conditions); agronomic efficacy not permissible

Note: FD2 corresponds to [15]; PD1 to [11]; PD2 to [13]; ND1 to [16]; Rubisco replacement to [8]. Numbers in eight-dimensional disclosure correspond to dimension numbers in Table 1; “reported” indicates minimum indicators for that dimension have been reported; “not reported” indicates a disclosure gap. *Arabidopsis* and tobacco are model plants; quality indicators in Dimension 7 are not required.

## Data Availability

All data generated or analyzed during this study are included in this published article.

## Supplementary Materials

The following supporting information can be downloaded at: https://www.mdpi.com/article/10.3390/biology15211964/s1, Table S1. risk-dependent module: additional reporting requirements; Table S2. claim-matching module: additional reporting requirements.
