# Supplementary material for: Photorespiratory Engineering from an Evolutionary Perspective: A Constraint-Aligned Evaluation and Validation Framework
Source: Biology (Basel). 2026 Nov 1;15(21):1964. doi: 10.3390/biology15211964 (PMC13761619; doi:10.3390/biology15211964)
Supplement: Supplementary file 1 [file biology-15-01964-s001.zip › biology-4659904-supplementary.pdf]

Supporting Information

Table S1. Risk-dependent module: additional reporting requirements.

Trigger condition	Additional mandatory reporting
Production of H_2O_2 in non-native compartment	Dimension 4: compartment redox state, H_2O_2 scavenging capacity, lipid peroxidation, photosystem damage and recovery
Inhibition of photorespiratory intermediate transport across chloroplast envelope (e.g., PLGG1)	Dimension 2: quantitative native/bypass flux partitioning, related intermediate accumulation levels
Direct suppression of GDC	Dimension 5: C–N–S coupling indicators, C_1 metabolism and SAM cycle, premature senescence and reproductive-stage phenotypes
Direct alteration of Rubisco catalytic center	Dimension 3: complete kinetic parameter set, Γ^*, chloroplast CO_2 estimates
Expression pattern covering reproductive stage	Dimensions 5, 6: reproductive-stage nitrogen reallocation, sink establishment and grain-filling dynamics

Table S2. Claim-matching module: additional reporting requirements.

Trigger condition	Additional mandatory reporting
Claiming agronomic value	Dimensions 6, 7: sink capacity, transport indicators, seed-setting rate, harvest index, yield components, quality indicators (food crops); grain crops must also report corresponding management conditions
Claiming stress adaptation	Dimensions 4, 6: compound stress experiments consistent with target cropping region and recovery dynamics
Claiming field deployability	Dimensions 7, 8: standard plot yield, multi-location, multi-year, multi-variety backgrounds, and variance decomposition
